# Electrical remodeling of atrioventricular junction: a study on retrogradely perfused chick embryonic heart

**DOI:** 10.1152/ajpheart.00115.2024

**Published:** 2024-07-19

**Authors:** Eva Zabrodska, Alena Kvasilova, David Sedmera, Veronika Olejnickova

**Affiliations:** Institute of Anatomy, First Faculty of Medicine, Charles University, Prague, Czech Republic

**Keywords:** atrioventricular accessory pathway, Langendorff perfusion, preexcitation

## Abstract

Atrioventricular (AV) accessory pathways (APs) provide additional electrical connections between the atria and ventricles, resulting in severe electrical disturbances. It is generally accepted that APs originate in the altered annulus fibrosus maturation in the late prenatal and perinatal period. However, current experimental methods cannot address their development in specific locations around the annulus fibrosus because of the inaccessibility of late fetal hearts for electrophysiological investigation under physiological conditions. In this study, we describe an approach for optical mapping of the retrogradely perfused chick heart in the last third of the incubation period. This system showed stability for electrophysiological measurement for several hours. This feature allowed analysis of the number and functionality of the APs separately in each clinically relevant position. Under physiological conditions, we also recorded the shortening of the AV delay with annulus fibrosus maturation and analyzed ventricular activation patterns after conduction through APs at specific locations. We observed a gradual regression of AP with an area-specific rate (left-sided APs disappeared first). The results also revealed a sudden drop in the number of active APs between *embryonic days 16* and *18*. Accessory myocardial AV connections were histologically documented in all positions around the annulus fibrosus even after hatching. The fact that no electrically active AP was present at this stage highlights the necessity of electrophysiological evaluation of accessory atrioventricular connections in studying AP formation.

**NEW & NOTEWORTHY** We present the use of retrograde perfusion and optical mapping to investigate, for the first time, the regression of accessory pathways during annulus fibrosus maturation, separately examining each clinically relevant location. The system enables measurements under physiological conditions and demonstrates long-lasting stability compared with other approaches. This study offers applications of the model to investigate electrical and/or functional development in late embryonic development without concern about heart viability.

## INTRODUCTION

Proper remodeling of the atrioventricular (AV) junction, leaving the sole electrical conduit within the fibrous heart skeleton, is critical for normal heart development. AV accessory pathways (APs) bypassing the annulus fibrosus provide an additional electrical connection. A functional AP results in abnormal ventricular activity such as in Wolf-Parkinson-White syndrome (WPWS), altered ejection fraction, atrioventricular reentry tachycardia (AVRT), or even life-threatening ventricular tachyarrhythmias in case of atrial fibrillation (1–6). The understanding of AP development at clinically relevant locations is thus of great importance. In WPWS, left-sided APs were predominantly reported (7–9). On the other hand, APs located in the right annulus or posteroseptally are more frequently found in children (10, 11).

During cardiac development, AV conduction in homeotherm vertebrates undergoes a substantial transformation. The replacement of a fully myocardial atrioventricular canal by a narrow AV bundle is revealed by the maturation of the ventricular activation pattern starting from the apex (12, 13). Although the morphological aspects of the annulus fibrosus formation are well documented, there is a paucity of data regarding the AV junction electrical remodeling. Previous studies relied mainly on histological observation (14, 15), which does not provide information about AP conductance (16). Therefore, functional data showing the electrophysiological characteristics of APs at specific positions around the annulus fibrosus are required.

Optical mapping is a technique allowing electrical activity visualization (17). With this approach, it is possible to determine the direction of impulse spreading and functionality of AP as a premature ventricular base activation. In the embryonic heart, optical mapping is traditionally used for early development up to the establishment of coronary circulation (18), becoming the primary source of nutrition (19). Unfortunately, the maturation of the annulus fibrosus and physiological AP regression take place during the postseptation period of heart development. At this stage, coronary circulation is fully developed. Hence, the heart viability is limited by the diffusion distance for oxygenation; this approach becomes unfeasible, and retrograde perfusion is critical for maintaining vital conditions (20, 21). Since histological data showed a similarity in the disappearance of accessory AV connections in the human and avian hearts (14, 15), it is possible to take advantage of the well-described embryonic cardiac development of the chick. Moreover, chick embryos offer favorable access and a more suitable heart size and aorta thickness compared with rodents (22, 23). Thus, this paper aimed to adapt the optical mapping technique for chick post-septated heart development. We used this approach to map the electrical remodeling of accessory AV connections during late embryonic and perinatal development. The results provide a better understanding of AP’s regression in a clinically relevant position.

## MATERIALS AND METHODS

### Animals

All procedures performed on animals were in accordance with the ethical standards of Charles University and were approved by the Animal Care and Use Committee of the First Faculty of Medicine.

Fertilized White Leghorn chicken eggs were incubated with continuous rocking to the desired prenatal day at 37.5°C, 45% humidity. The parameters inside the incubator were continuously monitored (W3811, Comet, Czech Republic).

### Heart Preparation and Perfusion

**Figure 1.**
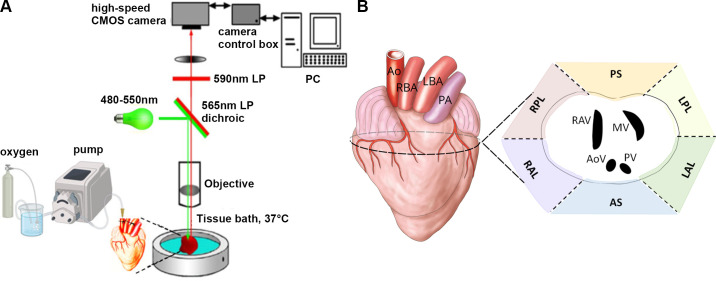
Experimental setup. *A* and *B*: schematic representation of the optical mapping of the perfused chick embryonic heart (*A*) and image of the isolated chick heart with representation of the atrioventricular (AV) junction and position of accessory pathways (APs) propagated on to the epicardial surface (*B*). Ao, aorta; AS, anteroseptal; LAL, left anterolateral; LBA, left brachiocephalic artery; LPL, left posterolateral, PA, pulmonary artery; PS, posteroseptal; RAL, right anterolateral; RAV, right AV valve; RBA, right brachiocephalic artery; RPL, right posterolateral. Image of fibrous heart skeleton adapted from Sedmera et al. (26). Figure was created with a licensed version of BioRender.com.

First, we used the nonperfusion approach on *embryonic day* (ED) *14* hearts (*n* = 6). The embryos were carefully removed from the egg and euthanized by cervical transection. To access the heart, a horizontal incision was made just above the xiphoid process, followed by a bilateral thoracotomy to open the body cavity. Careful dissection was then performed to remove the heart, ensuring it was not damaged. The hearts were then quickly placed in ice-cold Tyrode’s solution, consisting of (in mmol/L) 145 NaCl, 5.9 KCl, 1.1 CaCl_2_, 1.2 MgCl_2_, 11 glucose, and 5 HEPES (pH = 7.4), stained by voltage-sensitive dye di-4-ANNEPS (Thermo Fisher Scientific; 500 µL of 0.125% stock diluted in DMSO) for 10 min on ice (24) and mapped. We did not use any motion inhibitor in these hearts. We mapped these hearts with various temporal resolution (1–4 ms/frame). Since this approach proved insufficient to maintain the optimal condition in these hearts, we did not repeat measurements in the older chicks to follow the concept of 3 R and reduce the number of used animals. We, therefore, used a retrograde perfusion approach. We used embryos at the postseptated phase of embryonic development, particularly at the ED14 (*n* = 9), 16 (*n* = 15), and 18 (*n* = 13) and *posthatching day* (PH) *2* (*n* = 6). The aorta was cannulated under a dissection microscope (Nicon SMZ 800, Japan) with a custom-made cannula from a 25-gauge needle (B Braun). It is important to mention that significant differences exist in the mammalian and avian aortic arch morphology. Unlike mammals, the avian anatomy does not allow the cut under the first aortic branch, since the aorta branches a few millimeters above the aortic root (25) (Fig. 1). Therefore, the cannulation was performed on the ascending aorta, whereas the first two aortic branches (right and left brachiocephalic artery) were carefully ligated to avoid the solution leaking during perfusion. On the other hand, the thicker aortic wall in the chick embryonic heart allows cannulation, an approach not feasible in rodents at comparable developmental stages. After the cannulation, the heart was connected to the custom-made perfusion circuit and retrogradely perfused at a constant perfusion rate with Tyrode’s solution gassed with 100% oxygen at 37°C (Fig. 1). The perfusion rates were based on our previous measurement of the perfusion pressures during constant flow setting (ADInstruments, New Zealand) and set according to the reported pressure for similarly aged chick embryos (27). The specific perfusion rates were 0.6 mL/min for ED14, 0.8 mL/min for ED16, 1.4 mL/min for ED18, and 2.1 mL/min for PH2.

### Optical Mapping

To analyze the electrical remodeling of the AV connection in the post-septated heart development, we optimized our previously described retrograde perfusion setup connected to the optical mapping system (Fig. 1) and performed optical mapping on the post-septated heart embryonic heart. After 5 min of stabilization on perfusion, the hearts were stained with voltage-sensitive dye di-4-ANNEPS (with the same concentration as described above) by slow injection into the perfusion circuit for 5–7 min. To reduce the mechanical activity of the hearts, (+/−)-blebbistatin (Sigma-Aldrich, Cat. No. 203391) was then injected into the perfusion circuit to reach a final concentration of 14 µM. Imaging was performed using the ULTIMA L camera (Brain Vision, Japan) under a 2×, 0.14 NA objective lens (pixel size, 80 µm) with a water immersion lens cap (Olympus, Japan) on a fixed-stage BX51 WI epifluorescence microscope (Olympus, Japan) equipped with a 150-W Xe arc lamp (Cairn, UK) and an appropriate wide green filter set or under the THT Microscope (Brain Vision, Japan) with tandem Leica optics (28). Optical action potentials were recorded at the spontaneous rhythm from each heart’s anterior and posterior epicardial surface to obtain detailed information about AP localization. Sampling frequency was 1 kHz. To slow the conduction through the AV node and allow potential impulse propagation through slowly conducting APs, we applied adenosine at a concentration of 0.1 µmol/L, as was previously reported (16). After mapping, the hearts were fixed in 4% paraformaldehyde for 24 h and used for subsequent histological analysis.

### Data Processing

The data were band-pass filtered and processed using a 3 × 3 median filter to reduce noise. The first derivative was then numerically calculated, and its peak was used to detect pixel activation time. Spatiotemporal activation maps were then constructed as the isochronal maps in the BV_Ana software (Brain Vision, Japan), and the side of the first ventricular activation was detected based on the ventricular breakthrough. AV delay (AVD) was calculated from the signal delay between the first sites of atrial and ventricular activation.

### Localization of Electrically Active APs

The occurrence of APs and their particular location were analyzed according to their anatomical position (29). The propagation through the APs localized around the AV skeleton onto the epicardial surface was described as anteroseptal and posteroseptal, respectively. Other AP groups represent left anterolateral (LAL), left posterolateral (LPL), right anterolateral (RAL), and right posterolateral (RPL; Fig. 1).

### Histological Analysis

The hearts were rinsed in PBS and then dehydrated in a series of ethanols, followed by clearing in benzene, embedded in paraplast, and cut into 8-μm serial sections. To visualize the process of annulus fibrosus maturation, Picrosirius red (PSR; marks collagen I + III) and periostin staining were performed in adjacent sections. For PSR staining, sections were rinsed for 2.5–10 min (depending on the developmental stage) in a 0.1% PSR solution and immersed in a saturated picric acid solution (Sigma-Aldrich, Cat. No. 197378) for 5 min to enhance the yellow color of the working myocardium. For periostin staining, rehydrated sections were permeabilized with 0.5% Tween 20 and heat treated in citrate buffer (pH 6.0) for antigen retrieval. After cooling in 0.1% Tween 20, sections were blocked with 10% normal goat serum (NGS) and incubated overnight at +4°C with rabbit polyclonal anti-periostin (1:200, Abcam, Cat. No. ab14041) and mouse monoclonal IgG2b isotype to myosin heavy chain (MF20) antibody (1:10; DSHB, Cat. No. 2147781). Primary antibodies were detected using cyanine 5 (Cy5)-conjugated goat anti-rabbit IgG (1:200, Jackson ImmunoResearch, Cat. No. 111-175-144) and tetramethylrhodamine isothiocyanate (TRITC) goat anti-mouse IgG (1:200, Jackson ImmunoResearch, Cat. No. 115-025-146) secondary antibodies. The nuclei were counterstained with DAPI (1:1,000, BioVision, Cat. No. B1098).

Sections were imaged on an Olympus virtual microscope (slide scanner, ×20 objective) and Olympus BX61 with FluoView confocal system (Olympus, Japan) using ×40 and ×60 oil immersion objectives.

### Statistical Analysis

GraphPad Prism 9 (GraphPad Software, San Diego, CA) was used for graphic presentation and statistical analysis. The normality of data distribution was tested by the Shapiro–Wilk test. One-way ANOVA and subsequent Tukey test were used to compare differences in normally distributed variables between groups. Differences were considered statistically significant when *P* < 0.05.

## RESULTS

### System for Optical Mapping of Retrogradely Perfused Chick Embryonic Heart and Basal Electrophysiological Characteristic

**Figure 2.**
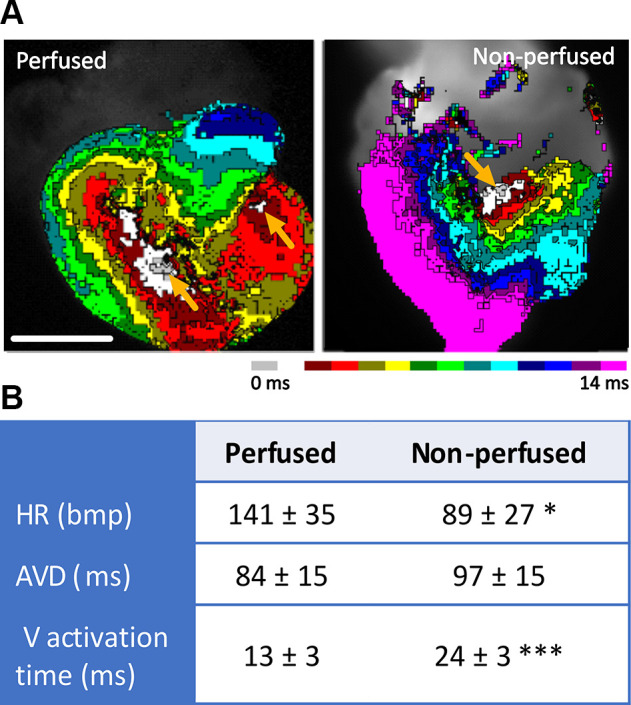
Nonperfused hearts show biased electrophysiological properties. *A* and *B*: representative optical map of the perfused and nonperfused chick *embryonic day* (ED) *14* hearts showing alteration of the electrical activation pattern (*A*), a significantly decreased heart rate, increased ventricular activation time, and the trend toward a longer atrioventricular delay in the nonperfused hearts (*B*). Scale bar = 2 mm. Data are expressed as means ± SD; *n* = 4 for nonperfused (beating hearts, total measured hearts were 6) and 9 for the perfused hearts; comparisons were assessed for statistical significance using a *t* test. AVD, atrioventricular delay; HR, heart rate; V activation time, ventricular activation time. *P < 0.05; ***P < 0.001.

To analyze the electrical remodeling of the AV junction throughout the postseptal heart development, we tested the ability of the optical mapping approach without the coronary perfusion relying on diffusion as a sole source of oxygenation. In the beating hearts (four hearts from six tested), we observed low heart rate (HR), implying low viability of the myocardial tissue (Fig. 2).

**Figure 3.**
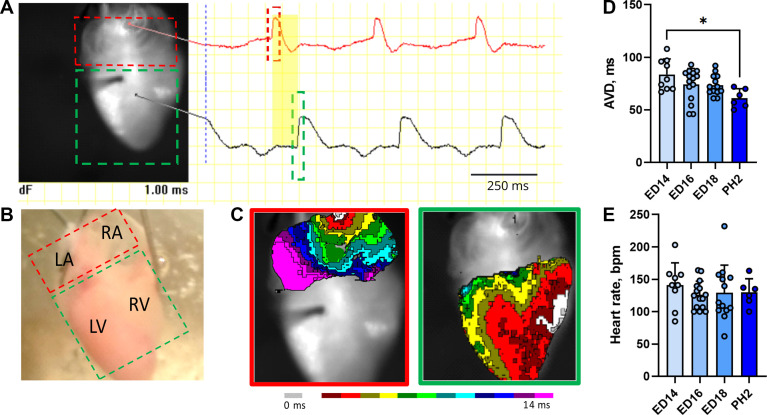
Basal electrophysiological characteristic of perfused late embryonic chick heart. Example of analysis from *embryonic day* (ED) *18* heart mapped from the posterior aspect showing a gray-scale image of the mapped area with traces corresponding to the action potentials from the atrium (red) and ventricle (black). *A*: yellow area shows the atrioventricular delay (AVD) in electrical signal propagation. *B* and *C*: photograph of the mapped area (*B*) and corresponding activation maps from the atria (red box) and ventricles (green box) (*C*). *D* and *E*: atrioventricular delay and heart rate of isolated chick ED14, ED16, ED18, and PH2 hearts; *n* = 6–15. Data are expressed as means ± SD; comparisons were assessed for statistical significance using one-way ANOVA with subsequent Student–Newman–Keuls test. *P < 0.05.

We therefore adjusted our retrograde perfusion system connected to the optical mapping to obtain physiologically relevant results. The process of optical mapping and subsequent analyses is summarized in Fig. 3. The activation was collected from the atria and ventricles with AVD (Fig. 3). This system proved stable for the chick embryonic heart for several hours without any changes in basal electrophysiological characteristics. Using this setup, we analyzed developmental changes in AV conduction as AVD, HR, and activation pattern. We observed a decrease in AVD between ED14 and PH2 (141 ± 35 vs. 130 ± 21 ms, *P* < 0.05; Fig. 3). The heart rate was not significantly different during all measured periods ranging from 141 ± 35 to 125 ± 21 beats/min (Fig. 3).

Direct comparison of both systems at ED 14 revealed significantly lower HR values, higher AVD, prolonged ventricular activation time, and altered activation patterns already during the first mapping in the nonperfusion approach (Fig. 2). These findings clearly indicate that the perfusion system is needed to obtain physiologically relevant results. Using this system, we also obtained activation maps for the atria and ventricles (Fig. 3, Supplemental Fig. S1; all Supplemental material is available at https://doi.org/10.6084/m9.figshare.25958335.v1). Typical atrial breakthrough occurred at the location of the sinoatrial node with subsequent impulse spreading along the crista terminalis and ultimately reaching both atria (Supplemental Fig. S1). Ventricular activation pattern corresponded with the terminations of the ventricular conduction system. Although activation through both bundle branches was clearly observed from the posterior aspect of the heart, the right bundle branch was dominant at the anterior cardiac aspect (Supplemental Fig. S1). There were no differences in both atrial and ventricular activation patterns across the analyzed developmental period. We recorded atrial and ventricular action potentials at all analyzed stages (Fig. 3). However, because of not fully suppressed motion artifacts, we did not measure the exact duration of the action potentials.

### Electrophysiological Parameters After Adenosine Injection

After mapping the basal condition, we perfused the heart with adenosine to reveal possible AP after the conduction through the AV node was slowed (16). The adenosine injection into the perfusion circuit immediately prolonged AVD (to 129 ± 17% at ED14, *P* < 0.05; 123 ± 24% at ED16; 129 ± 26% at ED18, *P* < 0.05, and 122 ± 18% at PH2) and decreased HR (to 71 ± 12% at ED14; 76 ± 19% at ED16, *P* < 0.001; 67 ± 17% at ED18, *P* < 0.001, and 67 ± 11% at PH2, *P* < 0.05). The effect of adenosine lasted for several tens of seconds, and after that, both AVD and HR returned to their baseline values.

### Persistence of Electrically Active APs During Late Embryonic Development

**Figure 4.**
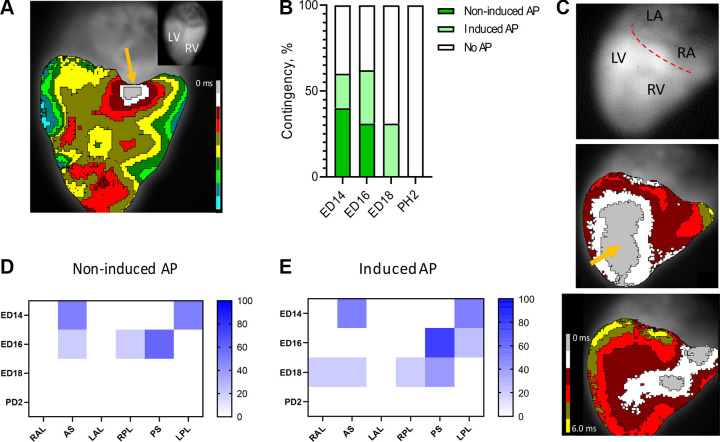
Localization of functional accessory pathways (APs) during late embryonic development. *A*: example of an epicardial activation map showing ventricular activation through an AP located in the septal posterior position (orange arrow). *B*: frequency of the noninduced and inducible AP in analyzed developmental stages. *C*: representative image of an inducible AP: gray scale of the mapped area (*top*), noninduced ventricular activation starting from termination of the His-Purkinje system at the apex (orange arrow; *middle*), and same heart after induction of activation through AP located at the right posterior location (orange arrow; *bottom*). *D* and *E*: frequency of noninduced (*D*) and induced (*E*) AP in all analyzed position. AS, anteroseptal; LA, left atrium; LAL, left anterolateral; LPL, left posterolateral; LV, left ventricle; PS, posteroseptal; RA, right atrium; RAL, right anterolateral; RPL, right posterolateral; RV, right ventricle.

The activation of the ventricle through APs was registered in the postseptated stages (Fig. 4). Such ventricular activation sequence starting from an AP was observed in both the ED14 and 16 (4 of 10, 40%, and 4 of 15, 31%, respectively; Fig. 4). On the other hand, no functional APs were detected without induction by adenosine in the older developmental stages, ED18 and PH2. In the ED18, conduction through an AP could be induced after AV node conduction slowing with adenosine (Fig. 4), but no APs were present in the PH2. Altogether, both younger stages were activated through noninduced or induced APs in more than half of the cases. On the contrary, functional AP propagation could be induced in less than half of the hearts in the ED18, and no AP conduction was detected or induced in PH2.

### Localization of Electrically Active AP During Late Embryonic Development

A septal position propagating through the anterior or posterior surface of the heart was the most frequent localization of APs (Fig. 4). Interestingly, we did not observe any left-sided AP active without adenosine induction from ED14 (Fig. 4). Similarly, no inducible left-sided AP was observed at ED18, where both right-sided and septal APs were still inducible (Fig. 4). In the last developmental stage where conduction through an AP was recorded, i.e., ED18, the most frequent location of APs was in the posteroseptal position.

### Morphological Persistence of Accessory Myocardial Continuity Through Annulus Fibrosus During Late Embryonic Development

**Figure 5.**
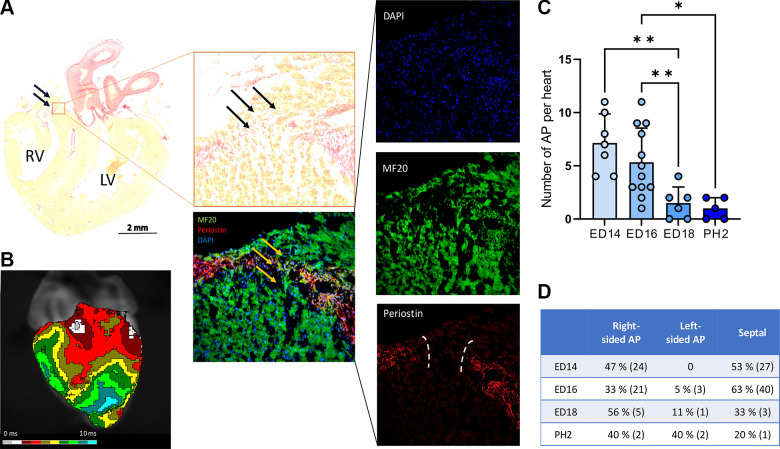
Persisting of accessory atrioventricular (AV) myocardial continuity within annulus fibrosus during late embryonic development. Picrosirius red (PSR) staining reveals the noncomplete fibrous heart skeleton with the passing myocardial strand. *A*: detailed view shows an accessory myocardial AV connection (MF20) within the periostin-positive annulus fibrosus. Nuclei were stained by 4′,6-diamidino-2-phenylindole (DAPI). *B–D*: optical activation map showing the conduction through the respective AP (*B*), numbers of APs per heart (*C*), and frequency (exact numbers in the parentheses) of AP positions within the AV connection (*D*). Scale bar = 2 mm. Data in *C* are expressed as means ± SD; comparisons were assessed for statistical significance using one-way ANOVA and subsequent Student–Newman–Keuls test. *P < 0.05; **P < 0.001.

Accessory myocardial AV continuity detected by optical mapping was morphologically detected in all analyzed stages (Fig. 5, Supplemental Fig. S2). However, their number decreased through development. In ED14, the number of connections was 7 ± 2, whereas only 1 ± 1 accessory myocardial AV continuity was present in PH2 (Fig. 5). The number of APs in ED16 was not significantly different from the one detected in ED14. Similarly, there was no significant difference in the number of APs between ED18 and PH2. Therefore, the main difference in number of morphologically detected APs was found between ED16 and ED18 (Fig. 5). Regarding the left-to-right position within the annulus, there was a similar pattern in the ED14 and ED16, showing the most abundant septal position of AP. However, for the older stages, the most frequent localization of APs was on the right side of the AV connection. The left-sided AP was less abundant during all stages (Fig. 5). Regarding the size of the accessory AV myocardial connections, we observed a decrease in the width of these myocardial strands from 53 ± 14 µm at ED14 to 28 ± 9 µm at PH2 (Supplemental Fig. S2). Interestingly, we did not observe any differences in the AP size based on their position (54 ± 13 µm for right-sided, 53 ± 2 µm for left-sided, and 58 ± 14 µm for septal AP, Supplemental Fig. S2). We do not provide the direct correlation between electrically active AP and accessory AV myocardial connections, as serial sectioning of the heart tissue may not capture some myocardial continuities accurately because of their curved nature. Furthermore, we have observed multiple myocardial connections within a single heart, particularly at earlier developmental stages. This complexity makes it difficult to ascertain which specific connection is responsible for observed electrical activity.

## DISCUSSION

We adapted an optical mapping system to study electrophysiological parameters in the chick embryonic heart. Using this system, we analyzed electrophysiological parameters in the hearts from ED14 (whole incubation takes 21 days). We used this approach to study the physiological AP regression during late embryonic development, where the optical mapping of the nonperfused hearts gives biased results due to suboptimal oxygenation and nutrition of the heart. Importantly, we were able to visualize ventricular activation patterns in the entire last third of the embryonic development.

Moreover, we monitored AP regression in the specific clinically relevant locations. This approach enabled direct visualization of AP location, mapping AVD, and analyzing activation patterns after the ventricular activation through AP, all in physiological conditions. Even though retrograde perfusion is a widely used experimental technique for various physiological, morphological, and pharmaceutical studies (21, 30, 63), its application in avian cardiovascular studies is highly restricted (31–34). We took advantage of the fact that chick hearts are considerably larger than prenatal and neonatal mouse hearts at equivalent stages. While mouse neonates weigh ∼1 g and rat pups weigh ∼6 g, newly hatched chicks weigh ∼17 g (35). Since we focused on the last third of the prehatching development and the presence of capillaries has already been demonstrated in its first third (18), establishing such a system was essential for maintaining vital conditions. Using this approach, we observed the gradual disappearance of both noninduced and induced APs. This process possesses an area-specific rate with the regression of left-sided AP in earlier development. Despite persisting AV myocardial continuity present after the hatching, no electrically active AP was present at this stage.

It is known that the transformation of the AV connection is not abrupt; rather, the myocardial connection gradually disappears (14, 15). Our longitudinal analyses showed that APs gradually decreased throughout the last third of the chick heart development. Interestingly, we observed a marked difference in the number of electrically active APs between the ED16 and ED18. We recorded activation through noninduced AP at ED16 and earlier, whereas no noninduced AP was detected at the ED18 and later stages. Moreover, conducting through AP, which contributed to the ventricular activation at half of the cases in the stage up to ED16, demonstrated a sudden drop in ED18. Histologically, accessory myocardial AV connections exhibited a similar decreasing trend through development. However, accessory myocardial AV connections were observed even after the hatching. This fact highlighted the necessity of a functional assessment of the APs in the developmental study of disorders such as preexcitation syndrome (e.g., WPWS) and/or AVRT.

To correlate the occurrence of APs with clinically relevant positions, we localized the occurrence of APs within the AV junction at each developmental stage separately. In the context of AVRT as a relatively common tachyarrhythmia in fetuses and neonates (10, 36), the successfully ablated simple APs were reported to be approximately one-third at posteroseptal localization (37). Similarly, our data showed posteroseptal position as the most abundant in the latest developmental stage, where conduction through AP was recorded, i.e., at ED18. Notably, the propagation through them was observed only after the conduction through the AV node was slowed. This feature assumes that they serve dominantly as the way for impulses to return to the atria in the AVRT called “orthodromic” AVRT. In this scenario, the AV conduction goes standardly through the AV node, and AP allows the impulse to return to the atria and close the arrhythmias circuit. This type of AVRT is more frequent compared with the reverse direction. Considering all analyzed stages, electrically active left-sided AP demonstrated a higher degree of disappearance during development than at the septal and right positions. Since the insulation in the human adult heart is usually poorly formed on the right side compared with the left part of the AV junction (38), it was speculated that there is a possible time difference in completing the right and left AV annulus (24). Notably, there are several reasons to anticipate that right-sided and septal APs might undergo some degree of spontaneous regression after birth. First, the observation that the prevalence of multiple APs decreases with the age of the child implies the spontaneous regression of some APs after birth (39). Second, compared with the situation in adults, APs located at the right annulus or posteroseptally are more frequent in neonates and young (10, 11). Third, apoptosis plays a role in perinatal heart development (39, 40), and it was associated with right-sided AP during development (24). Finally, it was revealed that most children with AVRT remain symptom-free without medication after the age of 1 yr (36, 41). As was mentioned before, in adults with WPWS, AP location was mainly described on the left side of the AV junction (42). Their fast conduction properties led to the assumption that these APs had different origins than those in the right and septal positions (43). We did not observe the noninduced AP after ED16, implying that their physiological regression was already in the earlier stages. This suggests that persistent left-sided APs in adults may result from a nonregression deficiency within early AV junction development. Intriguingly, a recent study has revealed a correlation of the epicardial fat volume with the inappropriate formation of AV electrical pathway and associated higher fat volume with first-degree AV block (44). Since subepicardial fat tissue represents a highly metabolically active tissue with paracrine activity (45), it could possibly be involved in AP formation and/or regression. When we considered all morphologically detected APs, not the functional ones only, they were found even after the hatching. A similar situation with the accessory myocardial strands connecting the atrium and ventricle was described in humans (46). In this context, it is important to consider the source-sink relationship, which necessitates a certain width of myocardial tissue to effectively depolarize downstream tissue (47). This principle, combined with our observation of a decrease in the width of accessory myocardial strands throughout development, contributes to the explanation of why not all morphologically detected accessory myocardial AV connections exhibited electrical activity.

In this article, we also analyze the AVD during the last third of chick prehatching development. We showed that in the isolated heart, i.e., without the neurohumoral modulation (30), the values continuously decrease with no distinguishable break after the hatching. Earlier studies reporting AVD in avian heart development performed measurements in suboptimal temperatures to meet metabolic demands without the necessity of coronary perfusion. This could have an important impact on the observed values (16, 48). Moreover, those studies used the quail heart; therefore, even a higher influence could be expected in chicks, considering the larger size and limited diffusion distance. Also, the measurement in nonperfusion mode significantly influences AVD values, as we demonstrated in this study.

Although we focused on the activation pattern, the optical mapping approach could also be used to determine the duration of action potentials. We have previously described differences in the shape and duration of atrial and ventricular action potentials in earlier embryonic stages (26). These differences are attributed to the distinct electrophysiological properties of the atrial and ventricular myocytes. The atrial myocytes exhibit a faster repolarization phase, contributing to their shorter action potential duration (31). When compared with the other vertebrates, the important differences in the action potential morphology could be recognized (49). The main variances exist within phase 2 of the action potential. In avian, long, thin cardiomyocytes lack transverse *t* tubules. Therefore, a strong transsarcolemmal Ca^2+^ influx via the L-type Ca^2+^ current and the high gain of Ca^2+^-induced Ca^2+^ release from the sarcoplasmic reticulum (SR), coupled with an internal SR Ca^2+^ release system, are needed to facilitate the contractions strong enough to meet the high metabolic rates of birds (50). This large influx of Ca^2+^ causes plateau in action potential described in avian hearts (51). On the other hand, in the mouse heart, the L-type Ca^2+^ current contributes less to the action potential; therefore, the murine AP shows a gradual repolarization rather than a distinct plateau phase (52).

The optical mapping of the retrogradely perfused embryonic heart could also be used to determine the atrial and ventricular activation pattern. Our experience showed that the posterior aspect is favorable for this purpose. When the atria are mapped from the anterior view, the great vessels impede the electrical recording of the middle atrial part. Similarly, the posterior aspect is better for mapping the ventricular electrical activity since the breakthroughs corresponding to both bundle branches are clearly visible. On the other hand, we typically observe the dominant activation through the right bundle branch when the mapping is performed from the anterior view. It could be speculated that this might be due to the faster activation through the moderator band. Alternatively, the faster right-sided activation could be attributed to the considerably thinner right ventricular wall in the birds compared with the left side of the heart. This right to left difference in ventricular wall thickness is higher than that in the mammalian hearts (53).

In conclusion, we present the system for optical mapping of the late embryonic chick heart to study the electrical remodeling of the AV junction. The retrograde perfusion of the coronary arteries was used to provide sufficient nutrition for measurements under physiological conditions. In this environment, we showed the gradual disappearance of the APs with an important drop between ED16 and ED18. We also described different rates of AP disappearance in clinically relevant positions around the annulus fibrosus, with the left-sided AP regression first. Finally, we demonstrated discrepancies between morphologically detected APs and their functionality, highlighting the necessity to include the electrophysiological evaluation of accessory myocardial AV connections in the study of AP formation. This approach provides the opportunity for other electrical or physiological measurements in late embryonic development.

### Limitations

Considering the limitation of the present study, only physiological remodeling of the AV junction was studied. Since we recently showed that the formation of the AV conduction axis is linked with the development of ventricular septation (46), the cardiac pathologies involving the altered forming of the cardiac crux are expected to be associated with a higher frequency of AP. Indeed, diseases such as Ebstein’s anomaly, Tetralogy of Fallot, and congenitally corrected transposition of the great arteries are linked with higher AP incidence (54–60). Therefore, we plan to further our study by modeling situations that mimic AP formation in the Tetralogy of Fallot (61) and ventricular septal defect (62). However, it is worth mentioning that conducting such investigations in mice, which are the dominant experimental model due to their suitability for genetic manipulation, is only feasible after birth. This is because their aortic wall lacks sufficient stiffness for cannulation, and the developed coronaries prevent diffusion from being the sole source of nutrition in late prenatal development. Despite these limitations, we believe that our data will help in better understanding AV junction remodeling in development and may elucidate the mechanism of the AP’s persistence in specific positions around annulus fibrosus.

## SUPPLEMENTAL MATERIAL

10.6084/m9.figshare.25958335.v1 Supplemental Figs. S1 and S2: https://doi.org/10.6084/m9.figshare.25958335.v1.

## GRANTS

This project has been supported by Czech Health Research Council Grant NU21J-02-00039; Charles University Cooperatio Grant 207029; Cardiovascular Science, Czech Science Foundation Grant 22-05271S; and National Institute for Research of Metabolic and Cardiovascular Diseases (Programme EXCELES, ID Project No. LX22NPO5104), funded by the European Union-Next Generation EU.

## DISCLOSURES

No conflicts of interest, financial or otherwise, are declared by the authors.

## AUTHOR CONTRIBUTIONS

D.S. and V.O. conceived and designed research; E.Z., A.K., and V.O. performed experiments; E.Z. and V.O. analyzed data; D.S. and V.O. interpreted results of experiments; E.Z., A.K., and V.O. prepared figures; E.Z., A.K., and V.O. drafted manuscript; D.S. and V.O. edited and revised manuscript; E.Z., A.K., D.S., and V.O. approved final version of manuscript.
